# GC-MS Analysis of Potentially Volatile Compounds of *Pleurotus ostreatus* Polar Extract: *In vitro* Antimicrobial, Cytotoxic, Immunomodulatory, and Antioxidant Activities

**DOI:** 10.3389/fmicb.2022.834525

**Published:** 2022-02-18

**Authors:** Doaa Hamad, Heba El-Sayed, Wafaa Ahmed, Hana Sonbol, Mohammed Abdel Halim Ramadan

**Affiliations:** ^1^Department of Microbiology and Immunology, Faculty of Pharmacy, Cairo University, Giza, Egypt; ^2^Department of Botany and Microbiology, Faculty of Science, Helwan University, Helwan, Egypt; ^3^Biochemistry and Molecular Biology Unit, Department of Cancer Biology, National Cancer Institute, Cairo University, Giza, Egypt; ^4^Department of Biology, College of Science, Princess Nourah Bint Abdulrahman University, Riyadh, Saudi Arabia

**Keywords:** *Pleurotus ostreatus*, antimicrobial, cytotoxicity, apoptosis, cell cycle, antioxidant, immunomodulatory, GC-mass spectroscopy

## Abstract

One strategy to manage resistant pathogens and develop potential anticancer drugs is the search for new, promising, and cost-effective medicinal benefits in the field of bioactive metabolites derived from mushrooms. In the current study, Egyptian cultivated *Pleurotus ostreatus* fruiting bodies polar extract was prepared to evaluate its antimicrobial activities as well as its cytotoxic effect on various cancer cell lines. The *Pleurotus ostreatus* polar extract (PoPE) was characterized by its phenolic and flavonoid content. The phenolics and flavonoids of PoPE were 6.94 and 0.15 mg/g, respectively. *P. ostreatus* polar extract showed potent antimicrobial activity against four pathogens, including *Candida albicans, Staphylococcus aureus*, *Micrococcus luteus*, and *Escherichia coli*. PoPE was found to inhibit *Fusarium oxysporum* (47%), *Fusarium solani* (28%) as well as *Rhizoctonia solani* (21%). PoPE was found to be 13 times more selective and toxic to MCF-7 cells than Vero normal cells, with the lowest IC50 value (4.5 μg/mL), so they were selected to examine the potential cytotoxic effects of PoPE. In MCF-7 cells, PoPE appeared to promote cell cycle arrest in the sub-G1 stage, as well as apoptosis. It significantly increased TNF-α production while decreasing IL-6 levels. PoPE’s total antioxidant capacity, lipid peroxide, and glutathione reductase activity were recorded 0.14 ± 0.02 mM/L, 15.60 ± 0.015 nmol/mL, and 9.50 ± 1.30 U/L, respectively. The existence of different bioactive metabolites was investigated *via* GC-MS, which confirmed the presence of 15 compounds with well-known biological activity.

## Introduction

Naturally occurring bioactive compounds are regarded as a rich source of many compounds with varying therapeutic potentials. Numerous natural compounds have been extracted from microorganisms, plants, and other living species to assess their potential as antimicrobial and antitumor alternatives and to investigate their diverse modes of action. Continuous attempts have developed numerous potential antimicrobial and antitumor treatments (Huang et al., 2021). Mushrooms have various dietary and medicinal benefits due to the presence of numerous bioactive compounds (Ha et al., 2020). Mushroom-derived bioactive metabolites were demonstrated to have antimicrobial, anticancer, immunomodulatory, anti-diabetic, antioxidant, anti-allergic, anti-inflammatory, and antiviral activities (Guggenheim et al., 2014; Elkhateeb, 2020; Venturella et al., 2021). The application areas of bioactive metabolites derived from mushrooms have switched to a new focus: the creation of innovative medications (Ha et al., 2020).

Antibacterial and anti-parasitic effects of *Flammulina velutipes*, *Ganoderma lucidum*, *Grifola frondosa*, *Lentinus edodes*, *Piptoporus betulinus*, *Schizophyllum commune*, *Trametes versicolor*, and *Volvariella volvacea* were reported for their therapeutic properties (Schillaci et al., 2013). Several studies have shown that certain mushrooms have cytotoxic or anti-cancer properties. According to Kim et al. (2017), Vulpinic acid, derived from *Pulveroboletus ravenelii* extract, inhibits cancer cells by inducing apoptosis. *Pleurotus eryngii* also contains several anticancer polysaccharides, and three pentacyclic triterpenoids with antitumor activity against MCF-7 breast cancer (Zhang et al., 2020). Mushroom polysaccharides have both immunostimulatory and immunosuppressive properties; the immunostimulatory property may be useful in cancer, while the immunosuppressive property may be useful in chronic inflammation (Ha et al., 2020). Mushrooms are high in phenolics, β-carotene, and vitamins, which are the primary contributors to their antioxidant action (Elhusseiny et al., 2021a).

The oyster mushroom (*Pleurotus ostreatus*) is a genus that is used not only as a food due to its flavor, aroma, and nutritional value, but also for its medicinal properties (Waktola and Temesgen, 2020). The genus *Pleurotus* contains approximately 40 species. *Pleurotus* is widely distributed in Egypt and The primary substrate used for *Pleurotus* spp. production in Egypt are chopped rice straw (Daba et al., 2008). Only a small percentage of fruit bodies sold in the Egyptian markets and they are always either *Pleurotus* or *Agaricus* mushroom (Shbeeb et al., 2019). It was reported that the maximum yield production of *Pleurotus* fruiting bodies were found during winter (14–27°C, and relative humidity at 70–80%) (Uddin et al., 2010). *P. ostreatus* extracts were shown to be antifungal (Chu et al., 2005), hypolipidemic (Alam et al., 2011), immunostimulant, antiproliferative, and antitumor (Abdel-Monem et al., 2020), as well as anti-inflammatory and antioxidant (Dkhil et al., 2020).

Egra et al. (2019) reported that water extract of *P. ostreatus* mycelium had the most significant cytotoxic potential by inducing apoptosis in human carcinoma cells. Furthermore, methanol extract of *P. ostreatus* fruiting bodies were found to have higher antioxidant, reducing power, radical scavenging, and iron chelating activities than other commercial mushrooms (Waktola and Temesgen, 2020). According to Okafor et al. (2017), hexane-dichloromethane extracts of *P. ostreatus* contain p-anisaldehyde, which inhibits pathogenic bacteria such as *Bacillus subtilis* and *Pseudomonas aeruginosa*, as well as pathogenic fungi such as *Aspergillus niger* and *Fusarium oxysporum*. Also, *P. ostreatus* basidia have antibacterial and antiparasitic properties. Akyuz and Kirbag (2009) investigated the antibacterial activity of *P. ostreatus* EVFB1 and EVFB4 extracts against bacteria such as *Listeria innocua*, *Bacillus cereus*, and *Escherichia coli*.

The main objective of this study was to investigate the antimicrobial, cytotoxicity, immunomodulatory, and antioxidant effects of the cultivated Egyptian mushroom *P. ostreatus* polar extract, as well as apoptotic induction and cell cycle arrest on cancer cell lines, followed by the extract’s volatile compounds identification by GC-MS technique.

## Materials and Methods

### Extraction of Mushroom Fruiting Bodies

**FIGURE 1:**
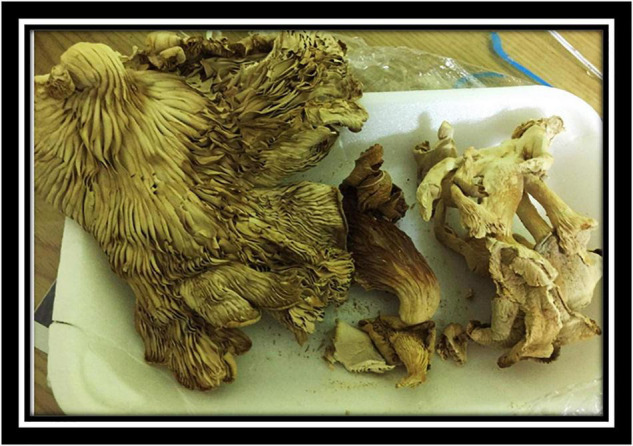
Fruiting bodies of *Pleurotus ostreatus*.

*Pleurotus ostreatus* was purchased from the Agriculture Research Center, Giza, Egypt. Fruiting bodies were crushed into a fine dry powder and kept in the refrigerator at 4°C for subsequent testing (Figure 1). Twenty grams of powdered *P. ostreatus* fruiting body were mixed with three solvents: methanol, chloroform, and distilled water, in a constant ratio of 2:2:1. Thereafter, the upper part of the extract (PoPE) was removed and vacuum-dried (Dkhil et al., 2020). The following equation was used to calculate the percent of extraction yield (Dokhaharani et al., 2021):

% extract yield = (Extract dry weight/dry fruiting body weight) × 100.

### Quantitative Determination of the Polar Extract’s Total Phenolics and Flavonoids Content

The total phenolics of the extract were estimated according to Soliman and El-Sayed (2021). 2.5 mL of Folin-reagent Ciocalteu’s diluted in 1:10 ethanol and 2 mL of 7.5% Na_2_CO_3_ were added to PoPE (0.5 mL). It was incubated at room temperature for 15 min before the sample absorbance at 765 nm was measured. Total phenolic content was estimated at mg of gallic acid equivalent (GAE) per g of dry extract using the gallic acid standard curve.

PoPE flavonoid content was quantified using the spectrophotometric assay with aluminum chloride. Briefly, 2 mL containing 0.1 mg/mL crude PoPE in methanol was mixed with 0.1 mL of 10% aluminum chloride and 0.1 mL of 0.1 mM potassium. After 30 min of incubation at room temperature, the absorbance of the mixture was measured at 415 nm. The quantified flavonoids were expressed as quercetin equivalents (QE) and were calculated in mg of quercetin per g of the dry extract (Mohammed et al., 2021).

### Screening for the Antimicrobial Effect

#### Microbial Growth Conditions and Reference Strains

PoPE was tested *in vitro* against some medically important bacterial species, such as Gram-negative bacterial pathogens (*E. coli ATCC 25922*, *Proteus mirabilis ATCC 29906*, and *P. aeruginosa ATCC 7853*), and Gram-positive bacterial pathogens (*Streptococcus pneumoniae ATCC 49619*, *Staphylococcus aureus ATCC 25923*, and *Micrococcus luteus ATCC 9341*) for growth inhibitory efficacy. At 37°C, the bacterial strains were routinely grown on a nutrient agar (Diffco) medium. Each organism was seeded with a single colony that was cultivated for 24 h at 37°C in a -nutrient broth medium. *Candida albicans ATCC 20231* was grown in potato dextrose broth medium for 24 h at 28°C as a pathogenic yeast. The antibacterial and anti-candidal activities for the investigated extract were evaluated using a well diffusion technique. The antifungal effect of PoPE on three pathogenic fungi (*F. oxysporum*, *Rhizoctonia solani*, and *Fusarium solani*) was measured based on mycelial growth inhibition percentage. The fungal pathogens were cultured for 3–5 days on a PDA medium at 25°C. All tested microbial cultures were obtained from the mycology lab at Helwan University, Faculty of Science in Egypt.

#### The Antibacterial and Anti-candidal Evaluation by Agar-Diffusion Technique

The agar diffusion method was used to test both antibacterial and anti-candidal activity (Avcı et al., 2014), in which 100 μL of the bacterial suspension and test yeast cultures (1 × 10^6^ CFU/mL) were spread into Petri dishes (9 mm) containing 20 mL of nutrient agar medium and 20 mL of PDA medium, respectively. To make wells, the agar plates were pierced using a cork borer with a 6-mm diameter. 100 μL of extract (20 mg/mL) was injected into the well independently by a sterile micropipette. The seeded plates were kept at 4°C for 8 h before being incubated for 24 h at 37°C. A negative control well containing 100 μg of water/methanol (2:1 v/v) was employed, whereas positive controls comprised antibacterial (gentamicin at 10 g/disc) and antifungal (amphotericin B at 100 units/disc) antibiotics. The plates were examined to see if an inhibition zone had formed around the wells. The antimicrobial action was determined by measuring the widths of the zones of inhibition (mm).

#### Screening of Antifungal Effect

The percentage of inhibitory effect on radial mycelial growth (PMGI) of three plant fungal pathogens (*F. oxysporum*, *R. solani*, and *F. solani*) was used to assess PoPE’s antifungal activity (Naglah et al., 2021). Potato dextrose agar medium (PDA) plates were loaded with 100 μL of the extract (20 mg/mL). After the diffusion of the extract into the agar, PDA fungal culture plugs (5 mm in diameter) were seeded into the plate. Untreated PDA plates from the same investigated fungi were used as a negative control. All cultures were cultured for 7 days at 25°C. The inhibition effect was evaluated by comparing the mycelium’s expansion radius on a PDA medium containing PoPE (R2) to that of the negative control media (R1). The PIMG was determined according to the formula below:


PIMG⁢=⁢{(R1-R2)/R1}⁢×⁢100.

### Evaluation of Cytotoxicity of PoPE

#### Cell Culture Conditions

In this investigation, cytotoxicity was evaluated in Vero, MCF-7, Hep-G2, Caco-2, and Hela cells supplied by the National Institute of Cancer, Egypt. These cell cultures were maintained in a 5% CO_2_ incubator and cultured in liquid RPMI-1640 medium with 10% (fetal bovine serum) FBS, 100 μg/mL streptomycin, and 100 units/mL penicillin at 37°C. The cell lines were sub-cultured regularly to maintain them in the exponential growth phase.

#### *In vitro* Assessment of Cell Viability and Cytotoxicity

To determine cell viability, the MTT test was applied. In a complete RPMI-1640 medium, cells were cultured (10,000 cells/well) into 96- well plates. Serial dilutions of the extract in physiological saline were made after 24 h to guarantee cell adhesion. For 24 h at 37°C in a humidified 5% CO_2_ environment, 100 μL of varied concentrations of PoPE (10,000–625 μg/mL) were applied. Following incubation, the culture medium was discarded, and 30 μL MTT solution/well was added and incubated for another 4 h. The developed color was detected at 570 nm by microplate ELISA. After constructing the dose-response graph with the GraphPad Prism 7 tool, the half inhibitory concentration (IC_50_) was determined. The cell viability percentage was determined using the formula:

The viability (%) = [Absorbance of treated wells/Absorbance of control wells] × 100.

#### Determination of PoPE Selectivity Index

The normal (Vero) cell line was used to estimate the selectivity index (SI). The SI of the extract was determined by dividing the toxic effect on the normal cell line by the toxic effect on cancer cell lines (Ogbole et al., 2017).

### Determination of Cell Cycle Using Cytell™ Cell Imaging System

MCF-7 cells were grown for 24 h at 37°C in RPMI-1640 medium supplemented with PoPE (5 μg/mL). The cell cycle was examined by the Cytell™ cell imaging system after staining with the Cytell™ Cell Cycle Kit (GE Healthcare, Tokyo, Japan).

### Apoptosis Analysis

MCF-7 cells (1 × 10^6^/well) were plated 24 h before the experiment and treated for 24 h with PoPE IC_50_ (5 μg/mL). Untreated cells were used to prepare the negative control. The kit for Apoptosis Detection (TACS^®^ Annexin V–FITC) was used to monitor apoptosis/live cells as specified by the manufacturer. The apoptotic/live cell percentage was determined by the Cytell™ cell imaging technique (GE Healthcare, Tokyo, Japan).

### Antioxidant Potential Assessment

PoPE (5 μg/mL) was cultured with MCF-7 cells for 48 h at 37°C. Colorimetric kits (Bio diagnostic, Giza, Egypt) were used to measure total antioxidant, lipid peroxide, and glutathione reductase according to the manufacturer’s guidelines.

### Determination of Cytokines (TNF-Alpha and IL-6) Levels by ELISA

TNF-α and IL-6 inflammatory cytokines were assessed in PoPE (5 μg/mL) treated MCF-7 cells grown at 37°C for 24 h in a 5% CO_2_ environment using an ELISA kit, according to the manufacturer’s guidelines (Sunlongbiotech, Hangzhou, China).

### Detection of Volatile Metabolites by GC-MS Technique

The volatile constituents of PoPE were examined by an Agilent 7000 Series Triple Quad Gas Chromatograph connected to a Mass Spectrometer. The conditions listed below were used: a capillary column (30 m × 0.25 m ID × 0.25 df); electron ionization at 70 eV; carrier gas (Helium 99.999%) with a flow rate of 1 mL/min; injector temperature of 250°C; an injection volume of 1 μL; inject split ratio of 1:10; and the ion-source temperature of 200°C. The oven was preheated to 110°C (isothermal for 2 min), then the temperature was raised at a rate of 10°C per minute up to 200°C, followed by a rise of 5°C/min to 280°C, and the final temperature was raised to 280°C for 9 min. At 70 eV, the spectral mass was acquired with a scan-interval of 0.5 s with fragment sizes ranging from 45 to 450 Da; the total time for the GC run was 36 min. Each component’s relative percent value was determined by calculating its peak value area to the total. The spectral data and chromatogram peaks were analyzed by the Turbomass program to determine the identity of the volatile compound.

### Statistical Analysis

The mean ± standard deviation (SD) was used to describe the data statistically. When comparing two groups, Student’s *t*-test was applied for independent variables, and when analyzing more than two groups, the *post hoc* multiple two-group comparison one-way analysis of variance test was used. Data is considered statistically significant at *p*-values < 0.05. IBM SPSS was used to perform all statistical calculations.

## Results and Discussion

### PoPE Yield

The polar extract productivity % (w/w) of *P. ostreatus* was 3.22%, as shown in Table 1.

**TABLE 1:** Total phenolics and flavonoids contents of *Pleurotus ostreatus* polar extract (PoPE).

Extract yield percent (W/W)	Phenolics (mg/g)	Flavonoid content (mg/g)
3.22%	6.94 ± 0.1	0.15 ± 0.1

*The results are presented in average ± standard deviations (SD) of three independent replicates.*

### Total Phenolics and Flavonoids Contents

The total phenolics were found to be 6.94 mg/g of GAE/g of dry PoPE (Table 1). González-Palma et al. (2016) evaluated polyphenols in *P. ostreatus* fruiting body extracts and observed that aqueous extract (11.36 mg GAE/g) revealed a higher content of phenolics than methanolic extract. Yim et al. (2009) found that the *P. ostreatus* water extract contains 7.23 mg GAE/g phenolic content. According to these findings, the total phenolic concentration of this Egyptian cultivated *P. ostreatus* polar extract was lower than previously reported.

In this research, flavonoids were found in low concentrations in PoPE, with 0.15 mg/g of *P. ostreatus* dry extract (Table 1). González-Palma et al. (2016) found that the flavonoid content of extracts from different stages of development in *P. ostreatus* was low, with no flavonoids detected in the fresh fruiting body and primordium water extracts. According to Gil-Ramírez et al. (2016), Mushrooms do not have flavonoids, so those identified in the extract could be owing to the absorbed nutrients and compounds from the medium in which they grow. In contrast, flavonoids were found in significant concentrations in ethanol extracts of *Pleurotus* species (Arbaayah and Umi Kalsom, 2013).

### The Antibacterial and Anti-candidal Activity Evaluation

The preliminary antimicrobial screening revealed that the PoPE possessed significant antimicrobial activity against some of the microbial pathogens tested, including, *C. albicans ATCC 20231*, *S. aureus ATCC 29213*, *M. luteus ATCC 9341*, and *E. coli ATCC 25922* (Table 2). Significant antimicrobial activity against *S. aureus* ATCC 25923 (i.e., 20 ± 0.7 mm) was observed, followed by *C. albicans ATCC 20231* (18 ± 0.1 mm), *E. coli ATCC25922* (16 ± 0.5 mm), and *M. luteus ATCC 9341* (12 ± 0.2 mm). In contrast, the tested *Streptococcus pneumoniae ATCC 49619*, *P. aeruginosa ATCC 7853*, and *Proteus mirabilis ATCC 29906* were all resistant to PoPE. It was reported that *P. ostreatus* methanolic extract had an antimicrobial effect against *E. coli* and *P. aeruginosa*, while *Pleurotus florida* had high antimicrobial activity against *E. coli* and *S. faecalis* (Gashaw et al., 2020). Methanolic extracts of *P. sajorcaju* demonstrated high bactericidal activity against *S. typhi* (MRD), according to Kandasamy et al. (2020). This discovery demonstrated that increasing extract concentrations (25–100 μg/mL) effectively confronted bacterial growth. According to Younis et al. (2015), the aqueous extract of the fruiting bodies of *P. ostreatus* had the strongest inhibitory efficacy against the tested yeasts *Cryptococcus humicola*, *C. albicans*, and *Trichosporon cutaneum*, while *S. aureus* and *E. coli* were the most sensitive test bacteria. Culture filtrate or biomass water extracts inhibited fungi and bacteria moderately. The obtained results are consistent with those presented by Vamanu (2012) regarding the ethanol mycelial extract of *P. ostreatus* PQMZ91109, which inhibits the *Candida* yeasts.

**TABLE 2:** Antimicrobial screening of *Pleurotus ostreatus* polar extract by the method of agar- well diffusion.

Microbial cultures	Inhibition zone diameter (mm)			
	*P. ostreatus* polar extract		Gentamicin (10 μg/disc)	Amphotericin B (100 units/disc)
Gram-positive bacteria	*Staphylococcus aureus ATCC25923*	20 ± 0.7^#^	21 ± 0.2	Nt
	*Micrococcus luteus ATCC 9341*	12 ± 0.2*	18 ± 0.1	Nt
	*Streptococcus pneumoniae ATCC49619*	–	15 ± 0.5	Nt
Gram-negative bacteria	*Escherichia coli ATCC25922*	16 ± 0.5*	25 ± 0.8	Nt
	*Pseudomonas aeruginosa ATCC 7853*	–	20 ± 0.6	Nt
	*Proteus mirabilis ATCC29906*	–	10 ± 0.0	Nt
Pathogenic yeast	*Candida albicans ATCC 20231*	18 ± 0.1*	Nt	22 ± 0.9

*The results are presented in average ± standard deviations of three independent replicates.*

*Indicates significance to standard antibiotics (P < 0.05).

*^#^Significance between the same group.*

*“–” Denotes no inhibition.*

*“Nt” Indicates Not tested.*

### Assessment of Antifungal Effect

**FIGURE 2:**
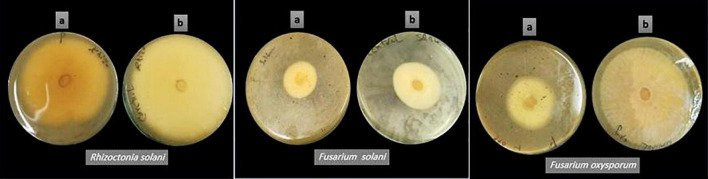
The mycelial growth-inhibitory influence of *Pleurotus ostreatus* polar extract on different pathogenic fungi (a) *P. ostreatus* polar extract-seeded plates, and (b) untreated control plates.

*Pleurotus ostreatus* polar extract was investigated for antifungal activity toward three plant pathogenic fungi. The crude extract’s antifungal effect against pathogenic fungi was determined using the percent inhibition of mycelial growth (PIMG), as shown in Table 3 and Figure 2. *P. ostreatus* polar extract had the highest (PIMG) value for *F. oxysporum* (47%), followed by *F. solani* (28%), and *R. solani* (21%). Four *Pleurotus* spp. mycelia were tested for antifungal activity against pathogenic fungi: *Verticillium* sp., *Trichoderma harzianum*, and *Pythium* sp., according to Owaid et al. (2017b). *Pleurotus salmoneostramineus* had the maximum inhibitory activity of 55.56 percent against *Verticillium* sp., while *P. ostreatus* mycelia had the lowest percent inhibition against *T. harzianum* of 46.15 percent. Owaid et al. (2017a) tested the antifungal efficacy of four oyster mushroom fruiting bodies taken from different agro-substrates. The extract of *Pleurotus cornucopiae* had the highest overall activity, while the lowest was found when cultivated on wheat straw. The highest inhibition activity was observed for *P. ostreatus* var. florida extract against *T. harzianum*, followed by *P. salmoneostramineus* extract against *Pythium* sp. and *Verticillium* sp.

**TABLE 3:** Antifungal effect of *Pleurotus ostreatus* fruiting body polar extract (PoPE).

	Percent of inhibition of mycelial growth (PIMG) %		
	*Rhizoctonia solani*	*Fusarium oxysporum*	*Fusarium solani*
Polar extract of *P. ostreatus*	21 ± 0.1	47 ± 0.2	28 ± 0.1

*Values for the PIMG percent are means ± SD of three independent replicas.*

### Evaluation of Cytotoxic Activity and Extract Selectivity

**FIGURE 3:**
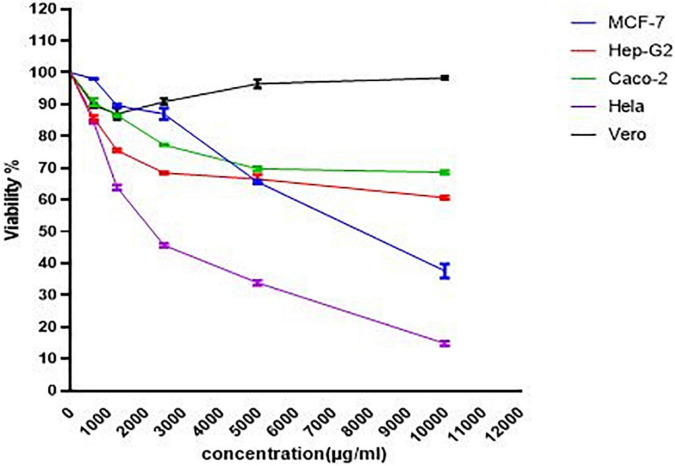
The viability dose-response curve for normal and cancer cells cultured with PoPE (10–0.625 mg/mL) for 24 h at 37°C. GraphPad Prism was used to calculate the results.

The cytotoxic potentials of PoPE were assessed in the different cultures of cancer and normal cell lines. Cell viability was tested after concentrations of 10,000 μg/mL were serially diluted down to 625 μg/mL and cell viability was revealed to be dose-dependent (Figure 3). *P. ostreatus* extract exhibited significant cytotoxic activity, with the lowest IC_50_ value for MCF-7 (IC_50_ 4.5 μg/mL), followed by Caco-2 (IC_50_ 25.4 μg/mL), HeLa cell line (IC_50_ 63 μg/mL), and *Pleurotus* polar extract was also tested on the growth of the Hep-G2 cell line after a 24-h incubation period to see if it had cytotoxic activity (IC_50_ 149 μg/mL), in comparison to the normal Vero cell line (60.4 μg/mL) (Table 4). Elhusseiny et al. (2021b) discovered that the fruiting bodies of *P. ostreatus* and *Lentinus edodes* water extracts decrease the survival of the tumor cell lines assessed by about 20%. Elhusseiny et al. (2021a) found that extracts of *Pleurotus sajor-caju* and *Agaricus bisporus* had the highest cell toxicity against the cell line, LS-513. According to Younis (2017), the antitumor activity of *Hericium erinaceus* extracts of mycelia and broth against the tested carcinoma cell lines was low, whereas the aqueous extract of fruiting bodies had the highest anticancer activity against MCF-7, Hep-G2, HeLa, and HCT 116 cells, with no effect on normal mouse hepatocytes. Younis et al. (2014) revealed that a water extract of *P. ostreatus* fruiting bodies had strong cytotoxicity against Hep-G2, HeLa, and HCT 116 cells.

**TABLE 4:** IC_50_ values and selectivity index (SI) of PoPE treated normal and cancer cells.

	Cell lines				
	Vero	MCF-7	Caco2	Hela	Hep-G2
IC_50_ (μg/mL)	60.4	4.5	25.4	63	149
Selectivity index (SI)		13.4	2.4	0.96	0.4
					

The SI data revealed that MCF-7 had a SI value of 13.4, Caco2 had a SI value of 2.4, Hela had a SI value of 0.96, and Hep-G2 had a SI value of 0.4. The primary purpose of medical treatment is to target cancer cells while minimizing harm to healthy cells. This remains a major obstacle to the use of various chemotherapy medications. As a result, selective toxicity must be taken into account when looking for new cancer treatment drugs. The SI of PoPE extract was assessed to see whether it would be cytotoxic to cancer cells while producing very low toxicity to normal cells (Wong et al., 2012). A SI of less than one is regarded as toxic, a SI of one to ten is considered weakly selective, and a SI of more than ten is considered non-toxic (safe) (Oluyemisi et al., 2015). In the current investigation, PoPE had a SI of 13.4, indicating that it was 13 times more toxic to MCF-7 cells than Vero normal cells. PoPE had a SI of 2.4 in Caco2 cells, indicating that the extract is only weakly selective for Caco2 cancer cells (Table 4).

Based on the previous results, IC_50_, and SI, MCF-7 cells had the lowest IC_50_, and the extract was highly selective for them. As a result, MCF-7 was chosen to investigate the possible cytotoxic effects of the tested extract.

### Cell Morphology and Cell Cycle Arrest by PoPE

**FIGURE 4:**
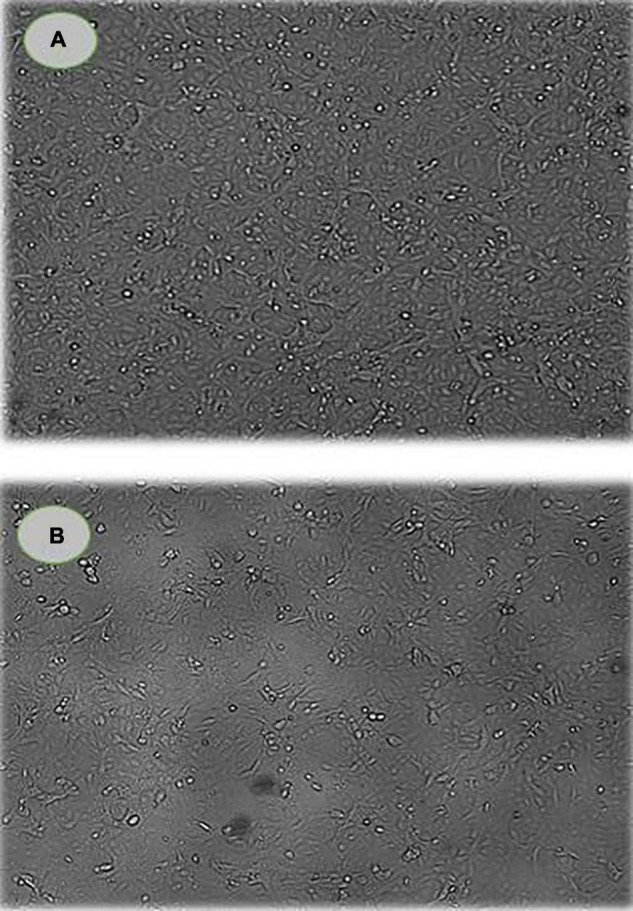
Cell viability and morphology of MCF-7 cells. **(A)** control cells; **(B)** PoPE-cultured MCF-7 cells (5 μg/mL). There was a significant cell size decrease and dead cells as a sign of apoptosis in treated cells.

The cell morphology and viability of MCF-7 cells were examined using the Cytell™ cell imaging system and the DAPI staining method after culturing with PoPE (5 μg/mL). PoPE-cultured MCF-7 cells had more round, shrunken, and blabbing membranes than untreated control cells. There were also dead cells and cell debris found (Figure 4).

**FIGURE 5:**
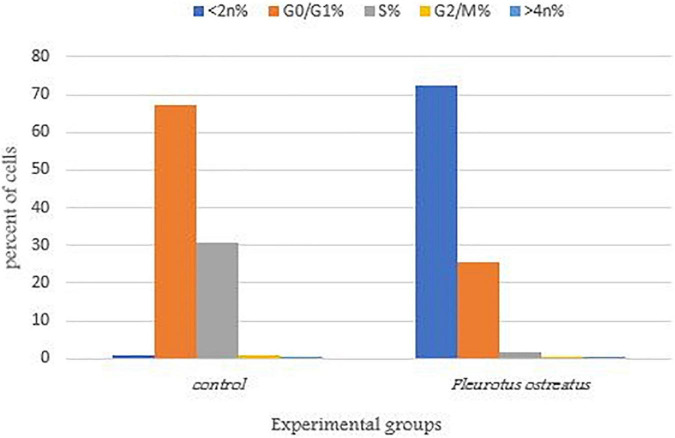
MCF-7 cells distribution in the various cell cycle stages treated with PoPE. The representative diagram showed induction of sub- G1 phase arrest in MCF-7 cells after incubation for 24 h and treatment with the IC_50_ (5 μg/mL) of PoPE as examined by a Cytell™ cell imaging instrument.

Cytotoxic activity can be caused by a variety of factors, including cell cycle arrest (Hsiao et al., 2007). To better understand the effects of PoPE on the MCF-7 cell cycle, the Cytell™ cell imaging system was used to evaluate the distribution of cell cycle phases. Figure 5 showed a representative histogram for MCF-7 cell cycle distribution that was exposed to PoPE. After cell treatment with PoPE (5 μg/mL), the cells % in the sub-G1 phase (apoptosis-inducing cells) increased significantly, reaching 72.62% compared to 0.79% in the control with a concomitant decrease in cells percent in the other cell cycle phases: G0/G1 (25.59%), S (1.65%), and G2/M (0.09%), when compared to control untreated cells (G0/G1 phase: 67.41%; S phase: 30.78%; and G2/M phase: 0.79%). PoPE, therefore, seems to be an inducer of sub- G1 phase cell cycle arrest after incubation for 24 h. A previous study showed that cell growth suppression of MCF-7 by *P. ostreatus* was linked to G0/G1 phase arrest (Jedinak and Sliva, 2008). It was reported that *F. velutipes* extract treated with plasma significantly induced the MCF-7 cells number in the G2/M phase arrest (Mitra et al., 2020). Ghosh et al. (2020) showed that ethanolic extract of *Hexagonia glabra* induced CaSki cells G2/M phase arrest. The G0/G1 stage arrest induction by *Pleurotus ferula* extract may inhibit cell proliferation in B16F10 cells, according to Wang et al. (2014).

### Apoptosis Induction

The presence of apoptotic cells in PoPE-cultured MCF-7 cells (5 μg/mL)was determined using a Cytell™ cell imaging system using an Apoptosis Detection Kit with Annexin V–FITC. Interestingly, our findings show that PoPE effectively induces apoptosis in MCF-7 cells. Common apoptotic characteristics include fragmentation of DNA, cell size shrinkage, and destruction of the cell membrane. These are examples of these processes observed in MCF-7 treated with PoPE as an indication of apoptosis induction (Figure 4).

**FIGURE 6:**
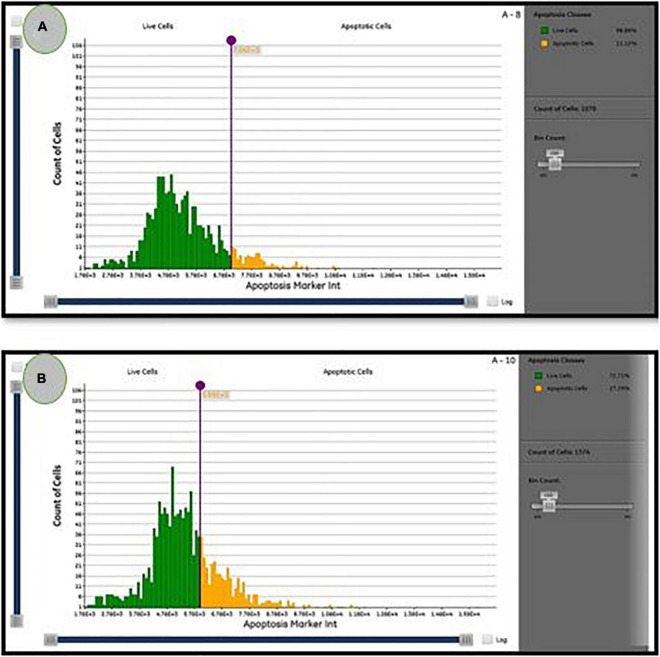
The percentage of live/apoptotic cells using the cytell™ cell imaging system. **(A)** Control cells; **(B)** PoPE treated MCF7 cells.

As illustrated in Figure 6, the percentage of apoptotic cells increased significantly to 27.3 ± 0.1% when compared with the untreated cells (11.12 ± 0.1%). According to Lavi et al. (2006), *P. ostreatus* water extract had cytotoxicity and induced apoptosis on HT-29 cells (Lavi et al., 2006). Mushroom extracts have the potential to be a successful therapy for breast cancer (Asatiani et al., 2011) and inhibit a range of cancer types, including mice hematological tumors and leukemia (Murakawa et al., 2007). Their mechanism of action is unknown, but apoptosis, over expression of genes in apoptosis-induction, and cell division inhibition are likely to be part of their mode of action (Fujimiya et al., 1998). Apoptosis is regarded as a preventive way against tumor proliferation by clearing neoplastic cells from the body (Murad et al., 2016). Following drug treatment in different cancers, apoptosis has been reported as the main mechanism of cancer cell death (Hickman, 1992). As a consequence, understanding apoptosis events and pathways may lead to the implementation of novel cancer treatment agents (Hengartner, 2000).

### Evaluation of Immunomodulatory Effect of PoPE

TNF-α is a pro-inflammatory cytokine with pleiotropic effects depending on its dose. The elevated concentrations cause a vasculotoxic impact, which is likely to be the cause of both its anti-cancer properties and the most life-threatening events (López-Legarda et al., 2020). Table 5 showed that TNF-α production increased significantly (*P* < 0.05) in cells of MCF-7 treated with 5 μg/mL PoPE (111.18 ± 0.01 pg/mL) compared to untreated MCF-7 cells (70 ± 0.2 pg/mL). In MCF-7 cells, TNF-α increases migration, invasion, and drug resistance by inducing apoptosis, inhibiting proliferation, and promoting apoptosis (Goldberg and Schwertfeger, 2010).

**TABLE 5:** Polar extract of *Pleurotus ostreatus* effects on IL-6 and TNF-α production in treated MCF-7 cancer cells and with negative control.

	Concentrations	
	Negative control	PoPE (5 μg/mL) treatment
TNF-α concentration (pg/mL)	70 ± 0.20	111.18 ± 0.01*
IL-6 concentration (pg/mL)	60.7 ± 0.82	35.623 ± 0.66*

The results are presented in average ± standard deviations of three independent replicates. *Statistical significance at P < 0.05.

IL-6 is one of the possible factors in breast cancer cell invasion and adhesion. The treatment of MCF-7 for 24 h with PoPE (5 μg/mL) showed a significant reduction in IL-6 production (*P* < 0.05). Table 5 showed that the concentration of IL-6 in untreated MCF-7 cells was 60.7 ± 0.82/mL, whereas MCF-7 treated with PoPE had a concentration of 35.623 ± 0.66/mL. It was reported that IL-6 enhances the MCF-7 breast cancer cell line’s development (Esquivel-Velázquez et al., 2015). Breast fibroblasts also release IL-6, which promotes MCF-7 cell proliferation and invasiveness (Baumgarten and Frasor, 2012). According to the findings of this research, the effects of PoPE on decreasing IL-6 as well as increasing TNF-α production in the MCF-7 cell line were suggested as a promising mechanism for immunomodulation and its cytotoxic activity. Mushroom components influence the immune system *via* many cellular pathways. Several genes are activated, resulting in a significant number of anti-inflammatory and anticancer cytokines (Ayeka, 2018). Previous research has revealed that *Lentinula edodes* enhance the secretion of cytotoxic and cytostatic cytokines and inhibit cancer cell proliferation and DNA synthesis in cells of breast cancer (Ayeka, 2018).

### Evaluation of Antioxidant Activity of PoPE

To determine the mechanism of PoPE’s cytotoxic action on the cells of MCF-7, the antioxidant activity (total antioxidant capacity, glutathione reductase, and lipid peroxide) was assessed. When compared to the control, MCF-7 cells with PoPE treatment at a concentration of (5 μg/mL) exhibited remarkable antioxidant activities. It had a non-significant reduction in total antioxidant capacity (0.14 ± 0.02 mM/L) when compared to untreated control MCF-7 cells (0.17 ± 0.03 mM/L). When compared to control (6.87 ± 0.38 U/L), glutathione reductase activity increased significantly in MCF-7 treated cells with a glutathione reductase concentration of 9.50 ± 1.30 U/L. When added to the MCF-7 cell line, the effect of PoPE (5 μg/mL) on lipid peroxide (MDA) level resulted in a marked decrease in MDA level. The MDA concentration was 15.60 ± 0.015 nmol/mL in the case of PoPE, compared to 21.70 ± 0.01 nmol/mL in the control (Table 6). According to previous data, lipid peroxidation could have a role in tumor formation because it produces reactive and toxic metabolites. MDA, as one of the most abundant and important lipid peroxidation aldehydes, can react with proteins, DNA, and other biomolecules, altering their structure and function (Kangari et al., 2018). Increased lipid peroxidation and oxidative stress are major contributors to breast cancer progression (Kangari et al., 2018). Glutathione acts as a reducing molecule during peroxide elimination by being oxidized and transformed into disulfide glutathione. Subsequently, the glutathione reductase enzyme regenerates glutathione by employing disulfide glutathione as a substrate. Glutathione reductase is activated in response to oxidative stress, and its activity is related to antioxidant status López-Barrera et al. (2021). The lower level of MDA detected in our study, as well as the increased activity of glutathione reductase, suggests that PoPE has strong antioxidant activity.

**TABLE 6:** The concentration of Total antioxidant, Lipid peroxide, and Glutathione reductase in PoPE’ MCF-7 treated group with the negative control group.

Antioxidants parameters	Concentrations	
	Negative control	PoPE (5 μg/mL) treatment
Total antioxidant (mM/L)	0.17 ± 0.03	0.14 ± 0.02
Glutathione reductase (U/L)	6.87 ± 0.38	9.50 ± 1.30*
Lipid peroxide (nmol/mL)	21.70 ± 0.01	15.60 ± 0.015*

The results are presented in average ± standard deviations of three independent replicates. *Statistical significance at P < 0.05.

### GC-MS Identification of the Volatile Constituents of PoPE

**FIGURE 7:**
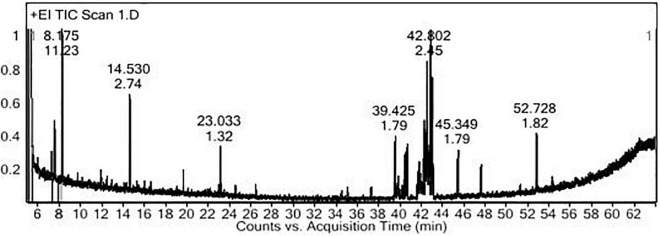
The polar extract of *Pleurotus ostreatus* fruiting body’s GC-MS chromatogram.

**FIGURE 8:**
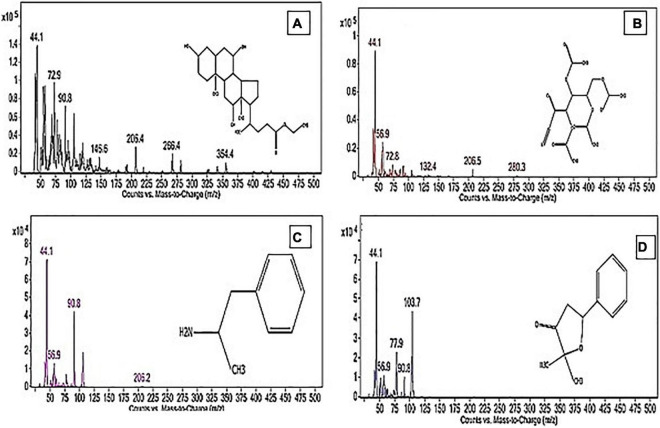
The structure of the principal chemicals found in *Pleurotus ostreatus* polar extract was revealed by the GC-MS chromatogram **(A)** Ethyl iso-allocholate, **(B)** Tetraacetyl-D-xylonic nitrile, **(C)** Amphetamine, and **(D)** 3(2H)-Furanone, dihydro-2,2-dimethyl-5-phenyl. The Supplementary Figure 1 has mass fragmentation patterns for the other detected chemicals that reported in Table 7.

The PoPE analysis results (Figures 7, 8) demonstrated the presence of 15 bioactive compounds. The peak area, retention time, and molecular formula were used to identify the phytochemical substances. Table 7 showed the compound name with its probability, area percent, retention time, and molecular formula. PoPE analysis revealed that Ethyl iso-allocholate was detected as a major compound by (62.5%), followed by 3(2H)-Furanone, dihydro-2,2-dimethyl-5-phenyl (11.23%), amphetamine (6.4%), Acetic acid, [(benzoyl amino)oxy] or Benzadox (2.74%), 7,8-Epoxylanostan-11-Ol,3-Acetoxy (2.45%), Toosendanin (2.06%), Flavone 4′-OH,5-OH,7-DI-O-Glucoside (2.01%), 1,3,2-Dioxaborolane,2,4,diethyl (1.82%), Benzaldehyde, 4-(dimethylamino) (1.79%), Pentacosan (1.79%), Tetraacetyl-D-xylonic nitrile (1.35%), Hexadecane (1.32%), 2-butenoic acid,2-methyl-2(acetyloxy)-1,1a,2,3,4,6,7,10,11,11a-decahydro-7,1 (0.95%), 2-Hexadecanol (0.9%), and Phytophylene (0.69%). Ethyl iso-allocholate is the main volatile component of steroidal derivatives, and it has been linked to anticancer, anti-inflammatory, and antimicrobial activities (Malathi et al., 2016; Neepal et al., 2019). It was reported that tetra acetyl-D-xylonic nitrile had antitumor and antioxidant properties (Hameed et al., 2015), whereas amphetamine belongs to the phenethylamine class and may have antidepressant and appetite suppressant properties (Biel and Bopp, 1978). Table 7 summarized the various known biological activities of the identified volatile bioactive compounds of PoPE. The chemical properties of the fresh and dried mushroom *P. ostreatus* grown on rice straw supplemented with wheat bran, as well as their bioactive secondary metabolic products, were studied using GC-MS. One hundred and seven metabolites were identified, including 2 acids, 5 alcohols, 27 alkane, 3 amides, 27 esters, 8 fatty acids, 4 terpenoids, 29 heterocyclic, and 2 phenols according to Mohamed and Farghaly (2014). González-Palma et al. (2016) reported that GC-Mass analysis of *P. ostreatus* fruiting body methanol extract indicated the existence of methyl tartronic acid (RT 20.185), which could be the antioxidant action source. According to Dkhil et al. (2020), HPLC analysis of *P. ostreatus* polar extract detected certain phenolics, including chlorogenic acid, that had antioxidant properties. Secondary metabolites and bioactive compounds identified by GC/MS in various mushroom and plants have previously been reported to have antimicrobial, anti-inflammatory, antioxidant, and antiproliferative activities (Riaz et al., 2012; Rizwan et al., 2012; Ashraf et al., 2014, 2018; Imran et al., 2014; Zubair et al., 2017; Majeed et al., 2021; Venturella et al., 2021). Therefore, the volatile bioactive components found *P. ostreatus* fruiting body polar extract may play important roles in the studied biological properties.

**TABLE 7:** Chemical profile identified from the *Pleurotus ostreatus* polar extract by GC-MS technique.

Compound name	Molecular formula	Molecular weight (g/mol)	RT (min)	Area sum%	Compound class	Biological activity
Ethyl iso-allocholate	C_27_H_48_O_5_	452.7	5.124	62.5	Steroidal derivative	Anticancer activity, anti-inflammatory (Neepal et al., 2019), Antimicrobial (Malathi et al., 2016).
Tetraacetyl-D-Xylonic Nitrile	C_14_H_17_NO_9_	343.29	5.439	1.35	-	Anti-tumor and antioxidant (Hameed et al., 2015).
Amphetamine	C_9_H_13_N	135.21	7.477	6.4	phenethylamine class	Antidepressant, and appetite suppressant (Biel and Bopp, 1978).
3(2H)-Furanone, –2,2-dimethyl-5-phenyl-	C_12_H_12_O_2_	188.22	8.175	11.23	Oxygen heterocyclic, functional group furanone	Anti-inflammatory action, treatment, and prevention of cancer (Semenok et al., 2019).
Acetic acid, {(benzoyl amino)oxy}, Benzadox	C_9_H_9_NO_4_	195.17	14.53	2.74	pesticide	Herbicide (Narwal et al., 2000).
Hexadecane (CAS)	C_16_H_34_	226.44	23.033	1.32	alkanes	Antifungal, antibacterial, antioxidant (Merr et al., 2019).
Benzaldehyde,4-(dimethylamino) or Benzaldehyde, p-(dimethylamino)	C_9_H_11_NO	149.19	39.425	1.79	benzaldehydes	The antitumor and antioxidant activities (el Bialy and Gouda, 2011).
2-Butenoic acid,2-methyl,2-(acetyloxy)-1,1a,2,3,4,6,7,10,11,11a-decahydro-7,10-dihydroxy-1,1,3,6,9-pentamethyl-4a,7a-epoxy-5H-cyclopenta[a]cyclopropa[f]cycloundecen-11-yl ester	C_27_H_38_O_8_	490.6	40.493	0.95	ester	No biological activity was reported.
Phytofluene	C_40_H_62_	542.9	40.597	0.69	carotenes	Reduce serum testosterone in prostate cancer, prevent breast and endometrial cancer, and has an antioxidant effect (Meléndez-Martínez et al., 2015).
7,8-Epoxylanostan-11- Ol,3-Acetoxy	C_32_H_54_O_4_	502.8	42.191	2.01	Alcoholic compound	No biological activity was reported
Hematoporphyrin	C_34_H_38_N_4_O_6_	598.7	42.802	2.45	organic compounds are known as porphyrins	Antitumor activity (Kim et al., 2003).
Toosendanin	C_30_H_38_O_11_	574.6	42.919	2.06	triterpenoid	Insecticidal activity (Guo et al., 2020).
Pentacosane	C_25_H_52_	352.7	45.349	1.79	Aliphatic hydrocarbon	Antibacterial (Konovalova et al., 2013).
2-Hexadecanol	C_16_H_34_O	242.44	47.517	0.9	long-chain fatty alcohols	Antioxidant (Merr et al., 2019).
1,3,2-Dioxaborolane,2,4-Diethyl	C_6_H_13_BO_2_	127.98	52.728	1.82	boronic esters	No reported activity-

*RT, Retention Time.*

## Conclusion

In conclusion, *P. ostreatus* polar extract demonstrated potent antimicrobial and antifungal activity against yeast, bacterial pathogens, and selected plant fungal pathogens. Furthermore, it appears to have a cytotoxic effect, as demonstrated in the studied cell lines, particularly breast cancer MCF-7. It inhibited growth, induced the arrest of a sub-G1 phase and apoptosis in breast MCF-7 cells. It directly stimulates TNF-α production while inhibiting the production of the inflammatory cytokine IL-6 in the MCF-7 cell line. This property of *P. ostreatus* extract qualifies it as a prospective immunomodulatory therapy for the management of many illnesses and cancer. The mushroom’s polar extract also reduced MDA levels and increased glutathione reductase activity, indicating its strong antioxidant potential. This mushroom, when consumed on diet, may provide protection against infectious diseases and cancer treatments by enhancing immunity. These findings are encouraging and open the way for additional research trials to find natural treatments for such serious diseases.

## Data Availability Statement

The original contributions presented in the study are included in the article/Supplementary Material, further inquiries can be directed to the corresponding author.

## Author Contributions

WA, MAR, and HE designed the study. HE and DH performed the experiments, prepared the fungal extract, and study its biological activity. DH and HS analyzed and wrote the results. DH wrote the draft of the manuscript. HE wrote and revised the final manuscript. WA, MAR, HS, and HE supervised and finalized the manuscript. All authors have read and agreed to the published version of the manuscript.

## Conflict of Interest

The authors declare that the research was conducted in the absence of any commercial or financial relationships that could be construed as a potential conflict of interest.

## Publisher’s Note

All claims expressed in this article are solely those of the authors and do not necessarily represent those of their affiliated organizations, or those of the publisher, the editors and the reviewers. Any product that may be evaluated in this article, or claim that may be made by its manufacturer, is not guaranteed or endorsed by the publisher.

## Abbreviations

GC-MS: gas chromatography-mass spectroscopy
MCF-7: breast cancer cell line “Michigan Cancer Foundation-7”
ELISA: enzyme-linked immunosorbent assay
TNF- α: tumor necrosis factor-alpha
IL-6: interleukin 6.
