# Supplementary material for: GC-MS Analysis of Potentially Volatile Compounds of Pleurotus ostreatus Polar Extract: In vitro Antimicrobial, Cytotoxic, Immunomodulatory, and Antioxidant Activities
Source: Front Microbiol. 2022 Feb 18;13:834525. doi: 10.3389/fmicb.2022.834525 (PMC8894875; doi:10.3389/fmicb.2022.834525)
Supplement: Supplementary file 1 [file Data_Sheet_1.pdf]

Supplementary Material

Supplementary Figures

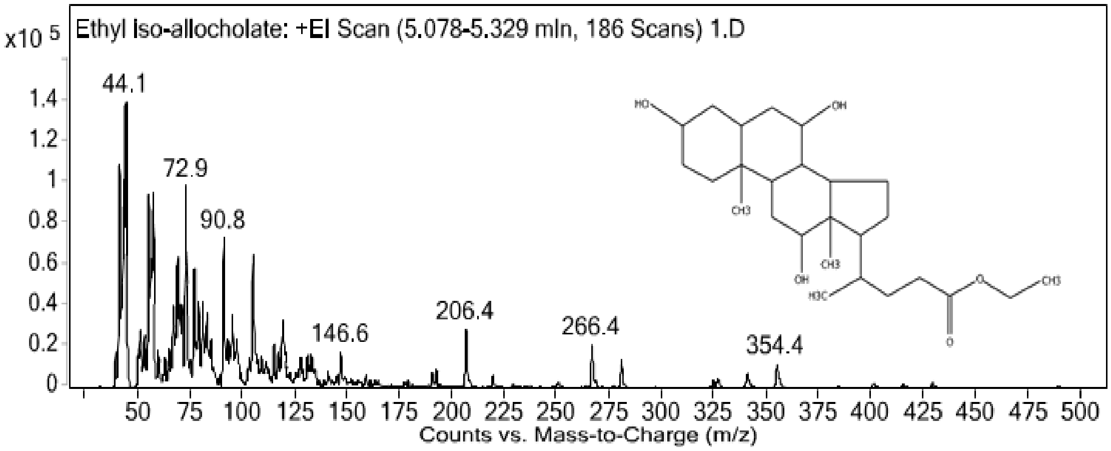

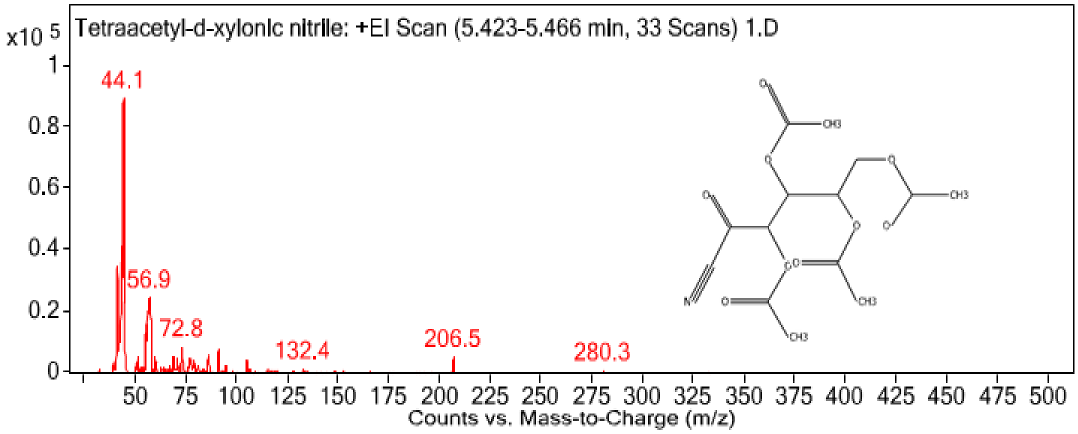

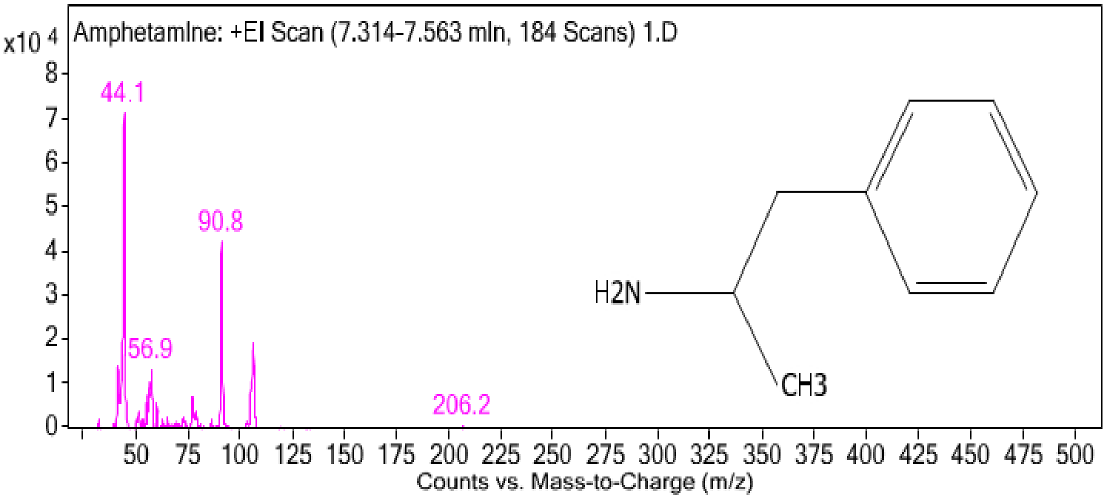

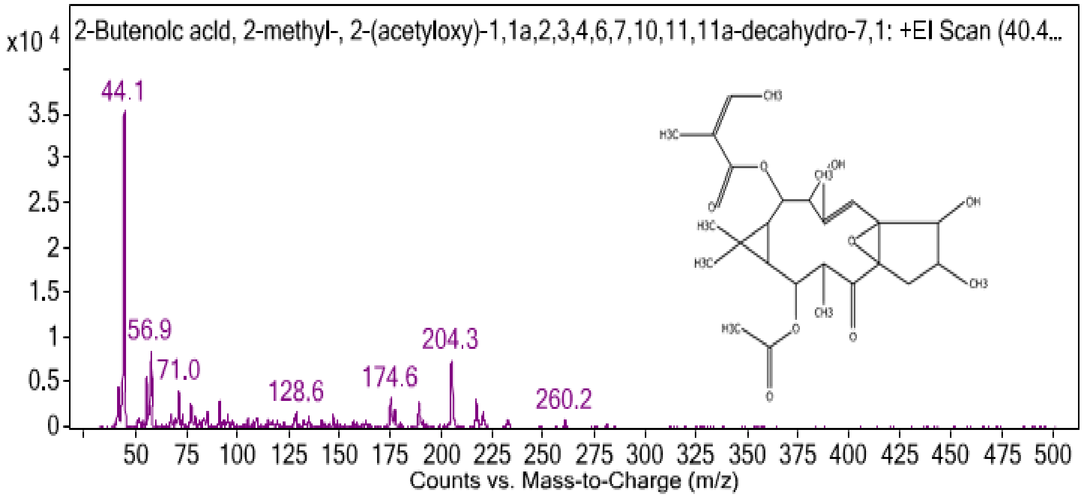

x10 4
2-Butenolc acld, 2-methyl-, 2-(acetyloxy)-1,1a,2,3,4,6,7,10,11,11a-decahydro-7,1: +EI Scan (40.4...
44.1
56.9
71.0
128.6
174.6
204.3
260.2
3.5
3
2.5
2
1.5
1
0.5
0
50 75 100 125 150 175 200 225 250 275 300 325 350 375 400 425 450 475 500
Counts vs. Mass-to-Charge (m/z)

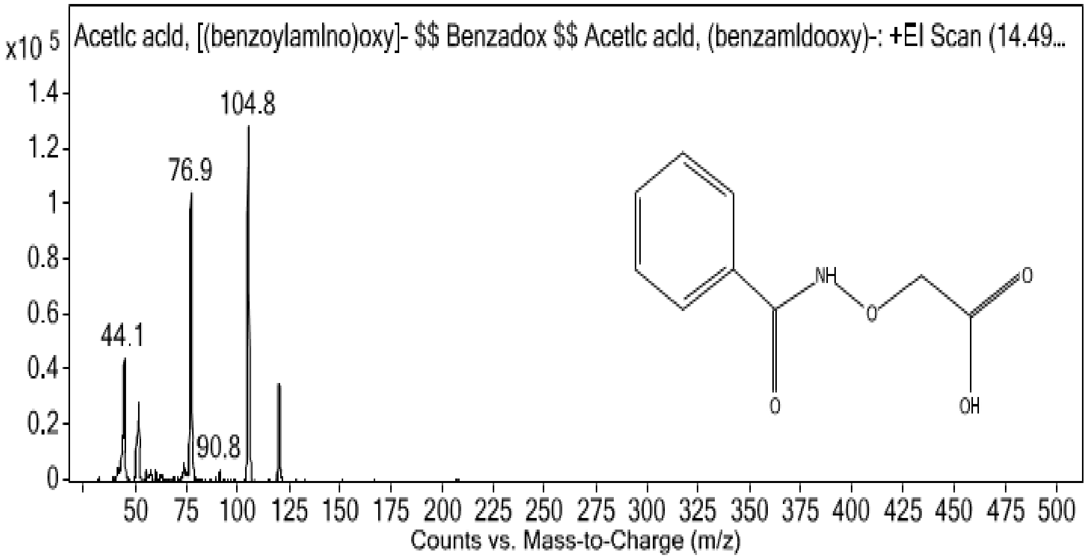

x10 5
Acetlc acld, [(benzoylamlno)oxy]- $$ Benzadox $$ Acetlc acld, (benzamldooxy)-: +EI Scan (14.49...
104.8
76.9
44.1
90.8
1.4
1.2
1
0.8
0.6
0.4
0.2
0
NH
O
OH
50 75 100 125 150 175 200 225 250 275 300 325 350 375 400 425 450 475 500
Counts vs. Mass-to-Charge (m/z)

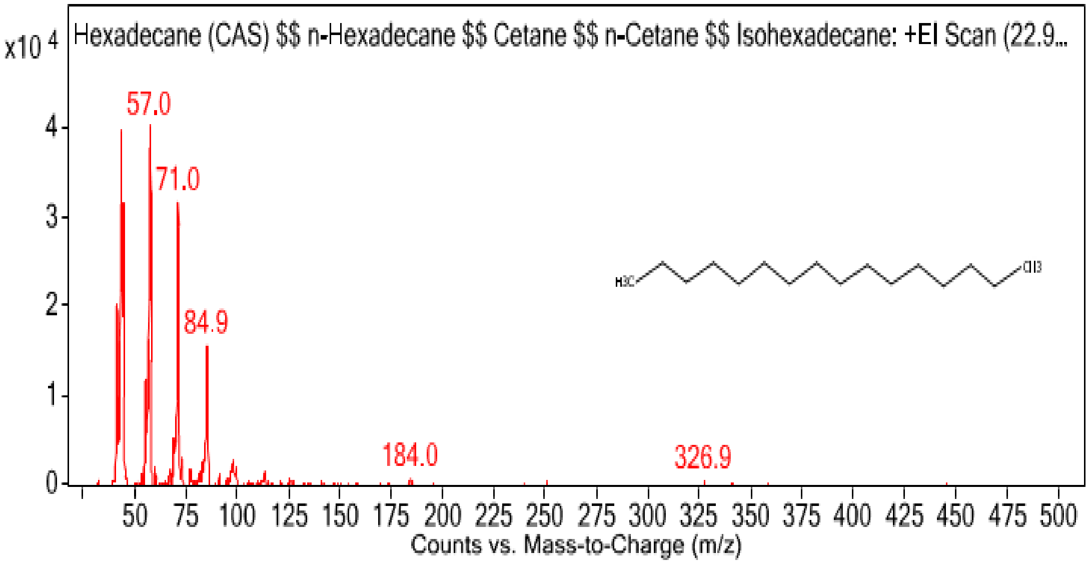

x10 4
Hexadecane (CAS) $$ n-Hexadecane $$ Cetane $$ n-Cetane $$ Isohexadecane: +EI Scan (22.9...
57.0
71.0
84.9
184.0
326.9
4
3
2
1
0
50 75 100 125 150 175 200 225 250 275 300 325 350 375 400 425 450 475 500
Counts vs. Mass-to-Charge (m/z)

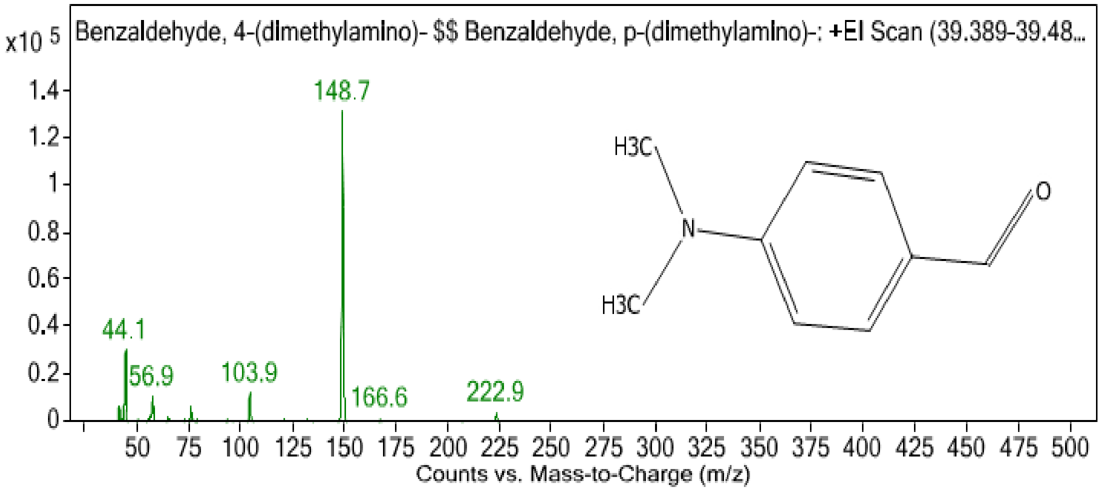
Benzaldehyde, 4-(dimethylamino)- $$ Benzaldehyde, p-(dimethylamino)-: +EI Scan (39.389-39.48...
x10 5
148.7
44.1
56.9
103.9
166.6
222.9
H3C
N
H3C
O
Counts vs. Mass-to-Charge (m/z)

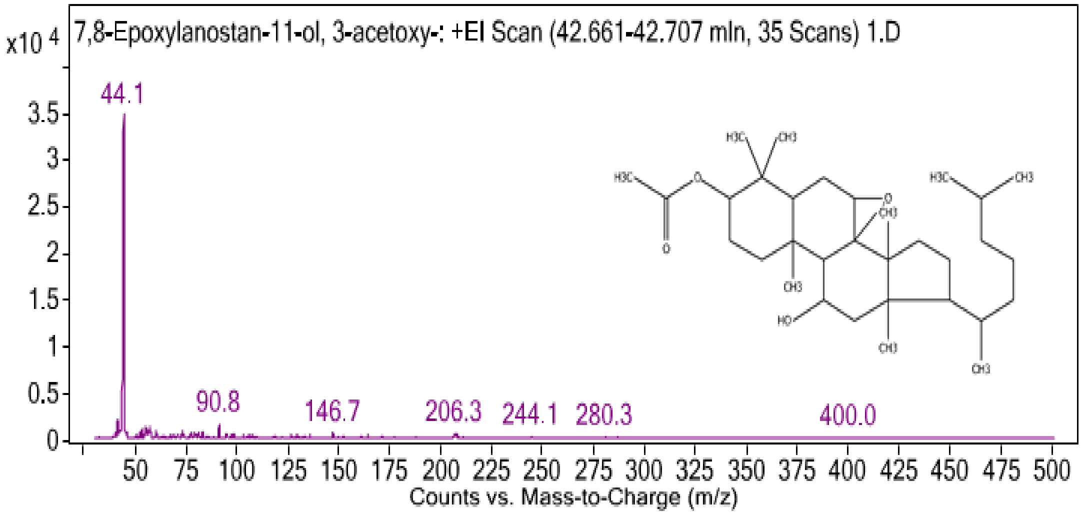
7,8-Epoxylanostan-11-ol, 3-acetoxy-: +EI Scan (42.661-42.707 min, 35 Scans) 1.D
x10 4
44.1
90.8
146.7
206.3
244.1
280.3
400.0
Counts vs. Mass-to-Charge (m/z)

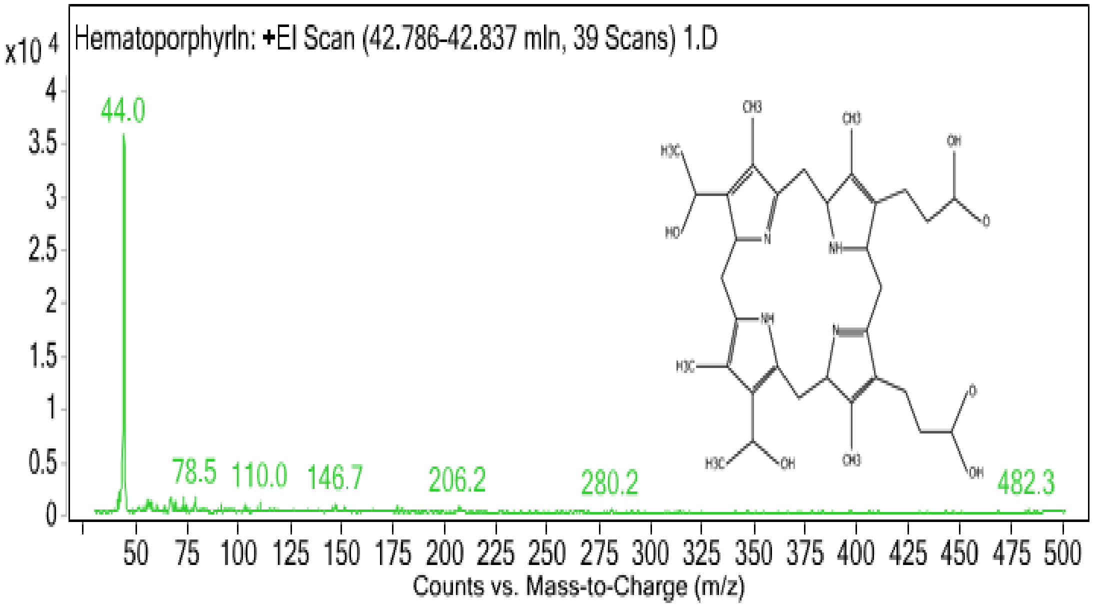
Hematoporphyrin: +EI Scan (42.786-42.837 min, 39 Scans) 1.D
x10 4
44.0
78.5
110.0
146.7
206.2
280.2
482.3
Counts vs. Mass-to-Charge (m/z)

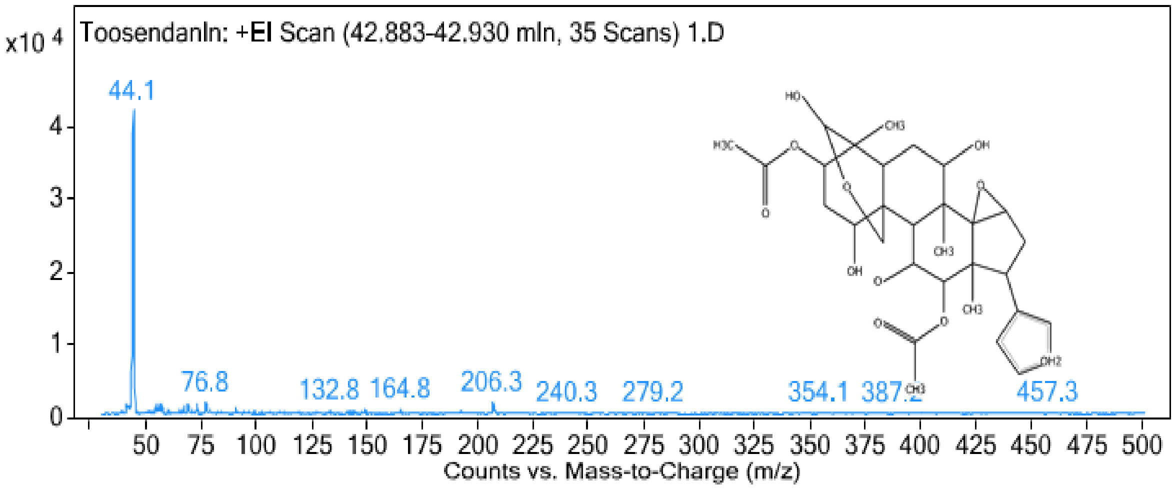
x10 4
Toosendanin: +EI Scan (42.883-42.930 min, 35 Scans) 1.D
44.1
76.8
132.8
164.8
206.3
240.3
279.2
354.1
387.2
457.3
Counts vs. Mass-to-Charge (m/z)

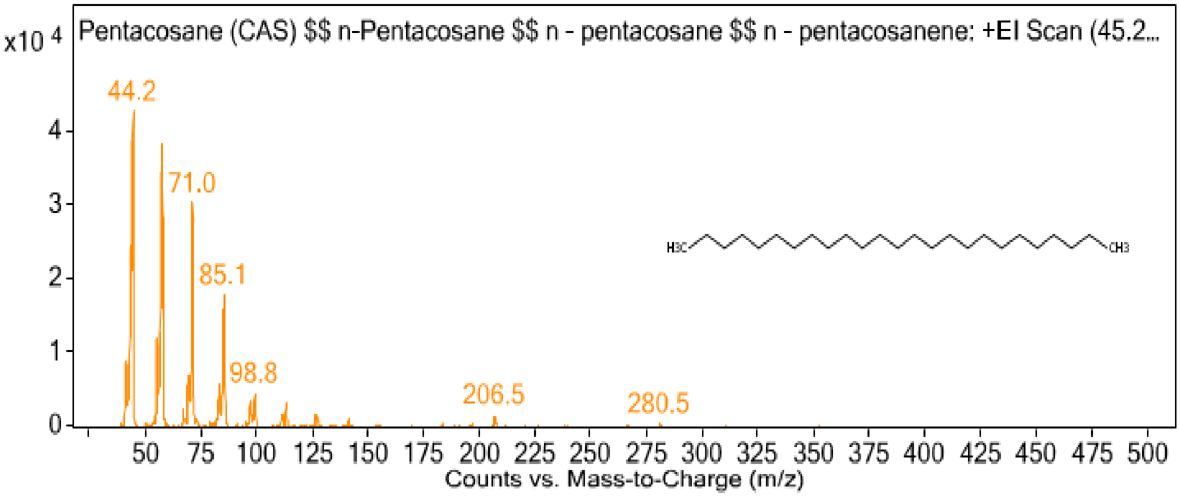
x10 4
Pentacosane (CAS) $$ n-Pentacosane $$ n - pentacosane $$ n - pentacosanene: +EI Scan (45.2...
44.2
71.0
85.1
98.8
206.5
280.5
Counts vs. Mass-to-Charge (m/z)

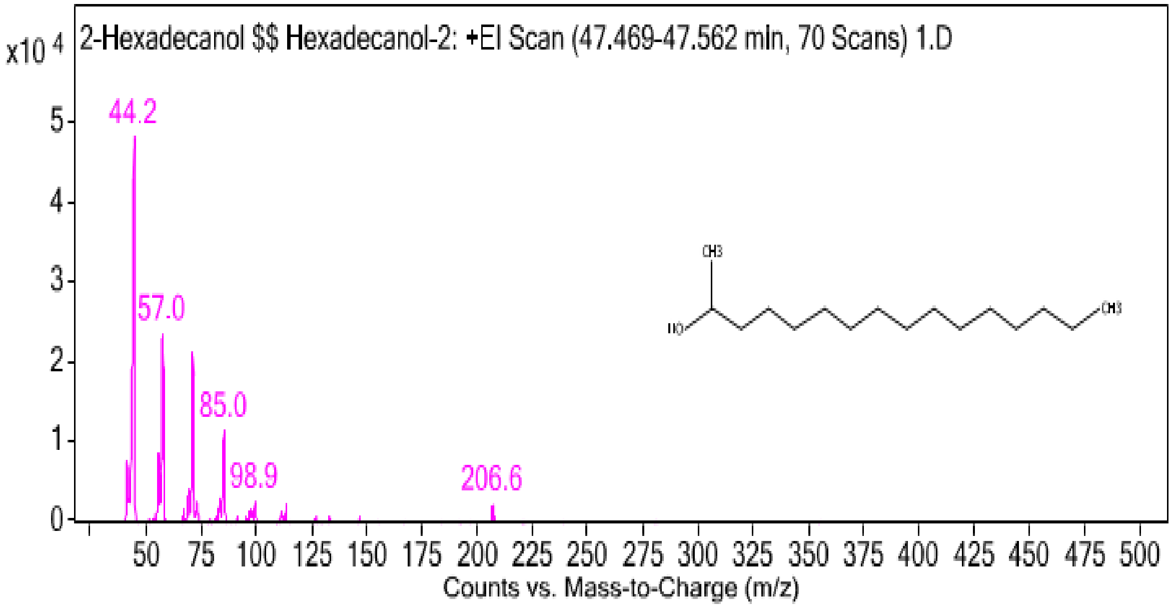
x10 4
2-Hexadecanol $$ Hexadecanol-2: +EI Scan (47.469-47.562 min, 70 Scans) 1.D
44.2
57.0
85.0
98.9
206.6
Counts vs. Mass-to-Charge (m/z)

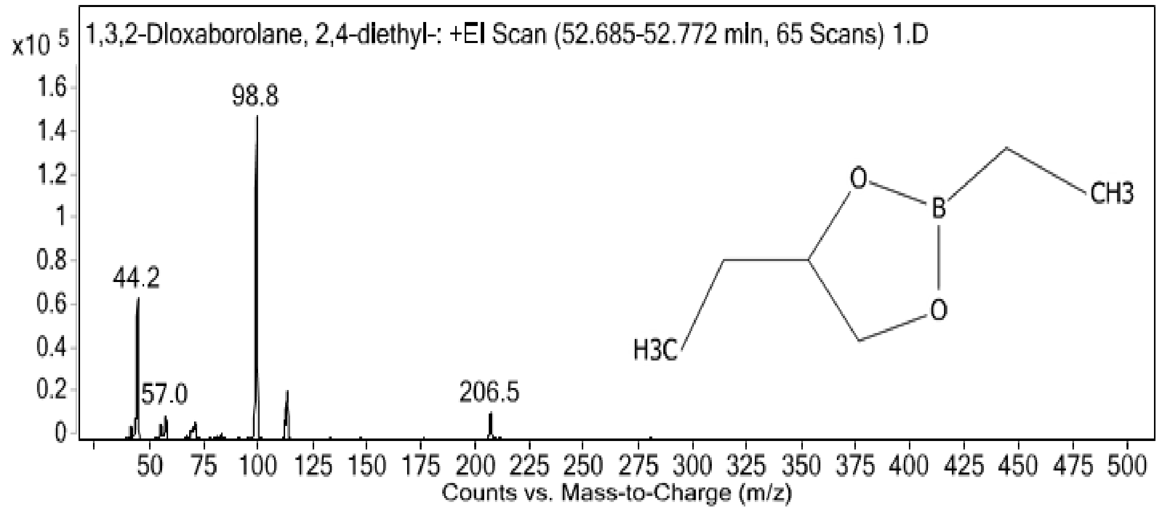

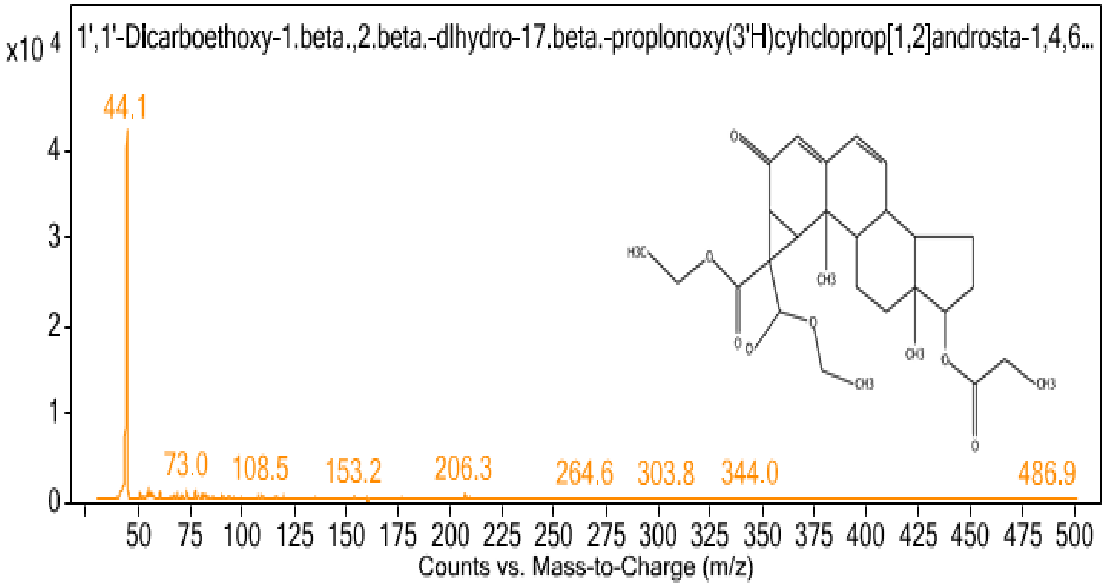

Supplementary Figure 1. The GC-MS chromatogram and the structure of the bioactive compounds identified in *P. ostreatus* polar extract
